# A Dynamic Noise Level Algorithm for Spectral Screening of Peptide MS/MS Spectra

**DOI:** 10.1186/1471-2105-11-436

**Published:** 2010-08-23

**Authors:** Hua Xu, Michael A Freitas

**Affiliations:** 1 Proteomics and Informatics Services Facility, University of Illinois at Chicago, Chicago, IL 60612, USA; 2 Department of Medicinal Chemistry and Pharmacognosy, University of Illinois at Chicago, Chicago, IL 60612, USA; 3 Department of Molecular Virology Immunology and Medical Genetics, Comprehensive Cancer Center, the Ohio State University Medical Center, Columbus, OH 43210, USA

## Abstract

**Background:**

High-throughput shotgun proteomics data contain a significant number of spectra from non-peptide ions or spectra of too poor quality to obtain highly confident peptide identifications. These spectra cannot be identified with any positive peptide matches in some database search programs or are identified with false positives in others. Removing these spectra can improve the database search results and lower computational expense.

**Results:**

A new algorithm has been developed to filter tandem mass spectra of poor quality from shotgun proteomic experiments. The algorithm determines the noise level dynamically and independently for each spectrum in a tandem mass spectrometric data set. Spectra are filtered based on a minimum number of required signal peaks with a signal-to-noise ratio of 2. The algorithm was tested with 23 sample data sets containing 62,117 total spectra.

**Conclusions:**

The spectral screening removed 89.0% of the tandem mass spectra that did not yield a peptide match when searched with the MassMatrix database search software. Only 6.0% of tandem mass spectra that yielded peptide matches considered to be true positive matches were lost after spectral screening. The algorithm was found to be very effective at removal of unidentified spectra in other database search programs including Mascot, OMSSA, and X!Tandem (75.93%-91.00%) with a small loss (3.59%-9.40%) of true positive matches.

## Background

Shotgun proteomics has gained increasing interest and become one of the most widely used tools in mass spectrometry (MS) based proteomics [1,2]. A large amount of data can be generated in high-throughput shotgun proteomics experiments. The analysis of these data presents many challenges. For example, high-throughput shotgun proteomics data contain a significant number of spectra from non-peptide ions or spectra of too poor quality to obtain highly confident peptide identifications. These spectra cannot be identified with any positive peptide matches in some database search programs or are identified with false positives in others [2]. Furthermore, these spectra consume data analysis time when searching the data set. Therefore, removing these spectra can improve the database search results and lower computational expense.

There have been many reports of algorithms used to filter poor quality tandem mass spectra. Moore *et al *developed an empirical model to assess the quality of tandem mass spectra prior to database search [3]. An apparent disadvantage of this model was that different selection criteria and different empirical parameters were needed for different mass spectrometers. Bern *et al *developed another algorithm to predict the quality of tandem mass spectra before database search. Their algorithm was able to filter 75% of the unidentified spectra of poor quality while keeping 90% of the identified spectra [4]. Wong *et al *reported an approach to assess spectral quality based on logistic regression using various spectral features [5]. This approach can be used to assess spectral quality and to filter poor quality spectra prior to database search. Purvine *et al *developed a spectral quality assessment method to filter tandem MS data prior to database search based on three features of a spectrum: 1) charge state differentiation, 2) total signal intensity, and 3) signal-to-noise estimates [6]. The noise in a spectrum was approximated to be the average intensity of the lower half of the peaks in the spectrum. The estimation can be heavily biased when too few or too many signal peaks of high abundance existing in the spectrum. Flikka *et al *used machine learning approach to differentiate poor quality spectra from good ones using various spectral feature of a tandem MS spectrum, including number of peaks, peak abundances and their standard deviation, precursor charge state, average *m*/*z *value and etc [7]. This method filtered up to 62% of unidentified spectra and was less efficient in filtering poor quality spectra compared to other methods.

Here we report a new dynamic noise level (DNL) algorithm, which is capable of filtering spectra of poor quality. The algorithm determines a noise level for each spectrum in a tandem MS data set by testing peaks from the lowest to highest. Based on that noise level, the algorithm then determines the number of signal peaks for the spectrum and its resulting quality. Poor quality spectra are excluded from further analysis. The algorithm was tested on a large tandem MS data set containing 62,117 spectra. Overall, the filtering achieved a significant reduction in false positives and unidentified spectra resulting in shorter database search times.

### Algorithm and Implementation

#### Dynamic Noise Level (DNL) Algorithm

For an experimental tandem mass spectrum with *N *peaks, the algorithm makes two assumptions about the tandem mass spectrum being filtered: 1) abundances of signal peaks are greater than those of noise peaks for the spectrum of good quality; 2) there is at least one noise peak in the spectrum due to electrical and/or chemical noise of the mass spectrometer. These two assumptions are reasonable for the mass spectrometers used for shotgun proteomics. All peaks in the spectrum are sorted according to their abundances, *I_i _*(*i *= 1, 2, ..., *N*), in an increasing order. Given the assumptions described above, the spectrum would consist of noise peaks followed by signal peaks. The first peak, i.e. the peak with the lowest abundance, is assumed to be noise. The algorithm then scans all peaks from *i *= 2 to *N *using the following algorithm until it finds the first signal peak:

1. For peak *k *being scanned, the previous *k*-1 peaks have been determined as noise by the previous scans. If *k *equals 2, the abundance of the second peak predicted by the first noise peak is calculated by the following equation,

(1) $${\hat{I}}_{2}=\left(1+\delta \right)\;I_{1};$$

where *δ *is a constant in the range [0,∞) that is dependent on the variation of ion abundances of noise peaks. *δ *equal to 0.5 is used in the current implementation of the algorithm.

2. if *k *is greater than 2, A linear regression model is fitted to the abundances of the previous *k*-1 sorted noise peaks,

(2) $$I_{i}=\alpha \;i+\beta ,\text{where }i=1,2,...,k-1\text{ and }k>2,$$

where *I_i _*is the abundance of peak *i*, *α *and *β *are linear regression parameters. Mathematically, the fitted parameters $\hat{\alpha }$ and $\hat{\beta }$ for the linear regression model are given by

(3) $${\left[\begin{array}{c} \hat{\alpha }\\ \hat{\beta } \end{array}\right]}_{2\times 1}={\left(A^{\text{T}}A\right)}^{-1}A^{\text{T}}I;$$

where, matrices $A={\left[\begin{array}{cc} 1 & 1\\ 2 & 1\\ \vdots & \vdots \\ k-1 & 1 \end{array}\right]}_{(k-1)\times 2}\;$ and $I={\left[\begin{array}{c} I_{1}\\ I_{2}\\ \vdots \\ I_{k-1} \end{array}\right]}_{(k-1)\times 1}$;

Under the hypothesis that peak *k *is noise, the abundance of peak *k *predicted by the previous *k*-1 noise peaks is given by,

(4) $${\hat{I}}_{k}=\hat{\alpha }\;k+\hat{\beta };$$

3. The signal-to-noise ratio (SNR) of peak *k *is estimated by the ratio of the observed peak abundance *I_k _*to the predicted peak abundance by assuming it is noise, i.e.

(5) $$\text{SNR}=\frac{{\text{I}}_{\text{k}}}{{\hat{I}}_{\text{k}}},$$

where ${\hat{I}}_{k}$ is calculated from equation 1 when *k *equals 2 and equation 4 when *k *is greater than 2. If the estimated SNR is greater than the threshold SNR_min_, peak *k *is determined to be signal and ${\hat{I}}_{k}$ is defined to be the noise level of this spectrum. Otherwise, it is noise and the scanning continues.

As a general rule of thumb, the minimum SNR for signal peaks in a tandem mass spectrum, SNR_min_, was set to be 2. The SNR threshold can be adjusted in more or less aggressive filtering as desired.

DNL algorithm can be used to screen tandem mass spectra of poor quality prior to database search. Based on assumption 1, all peaks with abundances greater than or equal to the first signal peak will be considered as signal. If the number of signal peaks, *n*, of the spectrum is below the threshold *n*_min_, the spectrum will be filtered. As a rule of thumb, the minimum number of signal peaks for a spectrum of good quality, *n*_min_, was set to be 8. The parameter *n*_min _can also be adjusted to allow for more or less aggressive filtering.

### Implementation

The DNL spectral screening algorithm described herein was implemented in a standalone program written in C++. The windows version of the program is freely available at http://www.massmatrix.net/download/. The algorithm is also incorporated in the MassMatrix database search engine for on-the-fly spectral screening during database search.

### Experimental

#### Sample Preparation and Mass Spectrometry

Bovine histones were isolated from bovine thymus tissue as described by Sures *et al *[8,9]. The mixture of bovine histones was digested by trypsin in 100 mM ammonium bicarbonate buffer (pH = 8.0). Enzymes were used in 25:1 ratio (substrate:enzyme) and the mixture was incubated at 37°C for two hours. The digested peptides were identified using data-dependent nano-LC-MS/MS on a LCQ Deca XP ion trap mass spectrometer (ThermoFisher, San Jose, CA, USA). 2.0 μL of bovine histone peptides with a total concentration of 0.1 μg/μL was injected into the LC-MS/MS system and eluted off the capillary HPLC column into the LCQ mass spectrometer using a linear gradient. Solvent A was water with 0.1% acetic acid and solvent B was acetenitrile with 0.1% acetic acid. Ions were fragmented by use of collision induced dissociation.

#### Database Search and Search Parameters

The RAW data files collected on the mass spectrometer were converted to MGF files and merged into a single large MGF file by use of MassMatrix data conversion tools http://www.massmatrix.net/download. The merged MGF file contained 62,117 tandem mass spectra. Tandem mass spectra that were not derived from singly charged precursor ions were searched as both doubly and triply charged precursors. Therefore, some spectra were searched with both +2 and +3 charges. This resulted in 86,147 tandem mass spectra in the data set to be processed and searched. The data set was filtered by the dynamic noise level algorithm. Both the original and filtered data sets were then searched by use of MassMatrix [10-12] (version 1.0.0, http://www.massmatrix.net against a protein database containing both the bovine histone database (117 proteins) and a decoy reversed human database (96,997 proteins) using the following options: i) No variable or fixed modifications; ii) Enzyme: trypsin; iii) Missed Cleavages: 3; iv) Peptide Length: 6 to 30 amino acid residues; and v) Mass tolerances of 2.0 Da and 0.8 Da for the precursor and product ions respectively. For each spectrum, the highest scored peptide match was assumed to be the best peptide hit.

The data sets were also evaluated by use of Mascot [13], OMSSA [14], and X!Tandem [15]. The counterpart search parameters in Mascot, OMSSA, and X!Tandem were identical to those in MassMatrix. For X!Tandem searches, refinement was enabled and performed for the peptide matches with expectation values greater than or equal to 1.0.

## Results and Discussion

**Figure 1:**
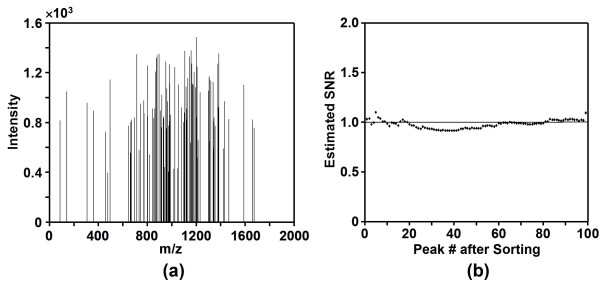
**Simulated noise spectrum**. (a) A simulated spectrum with 100 Gaussian noise peaks, and (b) the estimated signal-to-noise ratio (SNR) for all the noise peaks in it.

**Figure 2:**
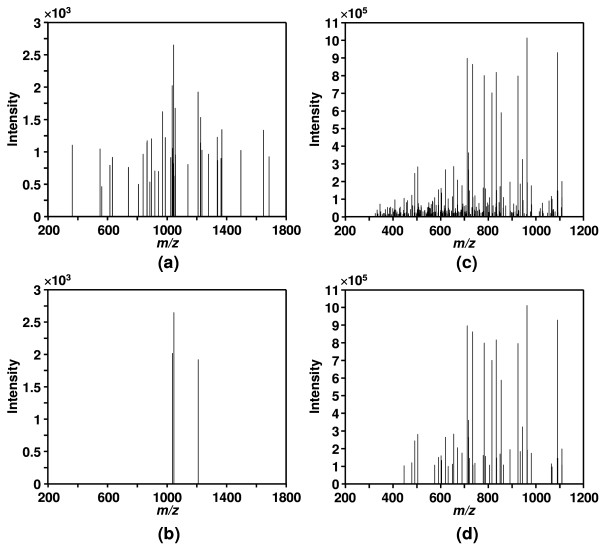
**Example noise and peptide tandem mass spectra before and after noise reduction**. An example tandem mass spectrum from a blank run (a) with 39 peaks before DNL noise reduction and (b) with 3 signal peaks after DNL noise reduction, and an example tandem mass spectrum for a peptide (c) with 279 peaks in it before DNL noise reduction and (d) with 46 signal peaks in it after DNL noise reduction.

The algorithm was first applied to a simulated noise spectrum containing 100 Gaussian noise peaks as shown in Figure 1a. The estimated SNR for the peaks are shown in Figure 1b. It can be seen that SNR for the peaks in the simulated noise spectrum were all close to the ideal value of 1 for noise peaks. Therefore, the algorithm successfully determined that all peaks were noise. The effectiveness of DNL spectral screening was tested by 4 real tandem MS data sets containing a total of 2232 tandem mass spectra from blank runs without injecting any peptide sample into the mass spectrometer. 99.15% of spectra in the data sets were successfully filtered by the DNL spectral screening algorithm. An example spectrum from the blank runs before and after DNL noise reduction is shown in Figures 2a &2b. It can be seen that the majority of the peaks in the spectrum were determined as noise and the spectrum was filtered by DNL algorithm as it only had three signal peaks. Figures 2c &2d show an example spectrum for a peptide before and after DNL noise reduction. The majority of the noise peaks were successfully removed from the spectrum and the number of total peaks was significantly reduced from 279 to 46. Furthermore, the spectrum was determined to be of high quality since it had 46 signal peaks.

The performance of the DNL algorithm was tested with a merged data set consisting of 23 shotgun proteomic experiments on bovine histone tryptic digests containing 62,117 total tandem mass spectra. The quality of the tandem mass spectra in the data set was evaluated by database searches in MassMatrix against a database containing a bovine histone database and a decoy reversed human database to eliminate the bias of manual evaluation. The decoy reversed human database created ~1000 times as many theoretical peptides as the bovine histone database. Therefore, false positive peptide matches from the bovine histone database were assumed to be negligible. The peptide matches returned from the bovine histone database were considered to be true positives (TPs), while those from the decoy database were, therefore, considered as false positives (FPs) [16]. The tandem mass spectra in the data set were classified into three categories: spectra identified with TPs, spectra identified with FPs, and spectra with no significant matches (unidentified spectra).

The effect of *δ *on spectral screening is ignorable due to the fact that majority of tandem MS spectra for peptides contain more than two noise peaks and the lowest noise peaks are not extreme compared to the higher noise peaks. For the merged data set containing 62,117 spectra from 23 experiments, the extreme setting of *δ *equal to 0 resulted in < 1% loss in sensitivity, i.e. the success rate of filtering bad spectra. The extreme setting of *δ *> 1.0 resulted in < 0.01% loss in specificity, i.e. the success rate of keeping good spectra. Therefore, a fixed intermediate setting of *δ *= 0.5 is used in the current implementation of DNL algorithm.

The different values for SNR and *n*_min _will affect the spectral screening results. The DNL screening algorithm at different SNR and *n*_min _settings was evaluated by use of receiver operating characteristic (ROC) analysis, i.e. sensitivity vs (1 - specificity) plots. The sensitivity of the DNL spectral screening is defined as

(6) $$\text{sensitivity}=\frac{\text{Number of correctly filtered spectra identified with no matches or FPs}}{\text{All spectra identified with no matches or FPs}}.$$

The specificity of the DNL spectral screening is defined as

(7) $$\text{specificity}=1\;-\;\frac{\text{Number of falsely filtered spectra identified with TPs}}{\text{All spectra identified with TPs}}.$$

Area under the curve (AUC) for the ROC curves indicates the overall discrimination power of the DNL spectral screening algorithm.

**Figure 3:**
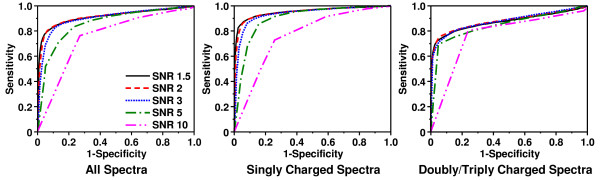
**ROC curves of the DNL spectral screening algorithm**. ROC curves of the DNL spectral screening algorithm with different SNR settings for the spectra with all charges, singly charged spectra, and doubly/triply charged spectra.

Figure 3 shows the ROC curves for the spectral screening algorithm with different SNR settings. It can be seen that the algorithm achieved high sensitivity and specificity with SNR between 1.5 and 3, which indicates its robustness over different SNR settings. A setting of SNR equal to 2 achieved the best overall discrimination powers with AUC values of 0.9149, 0.9474, 0.8792 for spectra with all charges, +1 charges, +2/+3 charges respectively. Therefore, it will be used for the discussion herein.

The ROC curve of SNR equal to 2 in Figure 3 is the sensitivity vs (1 - specificity) plot at various threshold settings of *n*_min_, i.e. the number of signal peaks. For singly charged spectra, a threshold *n*_min _equal to 9 has a specificity of 94.72% (i.e. false rate 5.28%) and a sensitivity of 85.67%. For doubly/triply charged spectra, a threshold *n*_min _equal to 7 has a specificity of 95.31% (i.e. false rate 4.69%) and a sensitivity of 73.69%. For all spectra, an overall threshold *n*_min _euqal to 8 achieved a specificity of 94.06% (i.e. false rate 5.94%) and a sensitivity of 80.07%. Applying different optimal *n*_min _thresholds for spectra with different charges provided very limited improvements with regard to sensitivities and specificities. Therefore, the current implementation of DNL algorithm does not support applying different settings for spectra with different charges. A setting of *n*_min _equal to 8 will be used for all spectra in the discussion herein.

The robustness of the DNL algorithm over different experiments was evaluated by the ROC analysis of the 23 individual tandem MS data sets as provided in the additional file [see Additional file 1]. It can be seen that the DNL algorithm achieved overall good power of discriminating good quality spectra from bad quality ones for all the data sets used.

**Figure 4:**
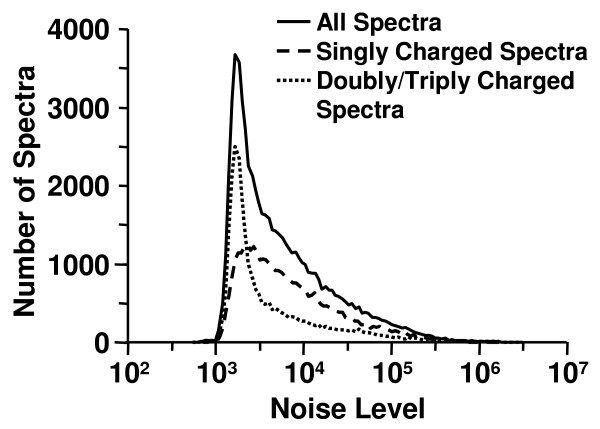
**Distributions of noise level determined by DNL algorithm**. Distributions of noise level determined by DNL algorithm for the spectra with all charges, singly charged spectra, and doubly/triply charged spectra.

Noise level represents the ion abundance of noise peaks in a spectrum. Noise level distributions obtained on the merged data set by use DNL algorithm with SNR setting equal to 2.0 are shown in Figure 4. It can be seen that noise level varied significantly from one spectrum to another over a range of 1000 to 1,000,000 in intensity. In addition, the data indicated that singly charged spectra tended to have a higher noise levels than doubly and triply charged spectra. The noise level distributions also varied from experiment to experiment.

**Figure 5:**
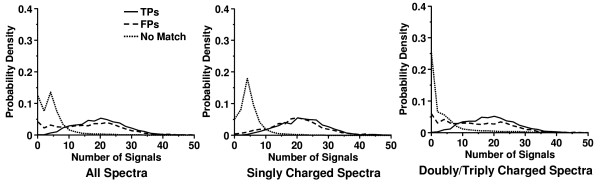
**Distributions of number of signal peaks determined by DNL spectral screening algorithm**. Distributions of number of signal peaks determined by DNL spectral screening algorithm for the spectra with TPs, FPs and the unidentified spectra. Peptide identifications were obtained from MassMatrix database search engine.

**Figure 6:**
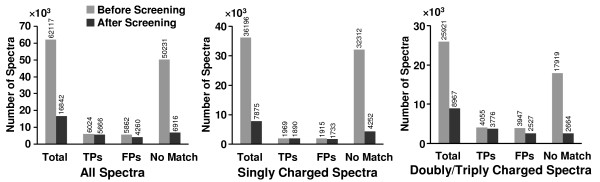
**Effect of DNL spectral screening on MassMatrix search results**. Histograms showing the effect of DNL spectral screening on tandem mass spectra for the spectra with TPs, FPs and the unidentified spectra. Peptide identifications were obtained from MassMatrix database search engine.

The number of signal peaks for each spectrum in the data set was also determined by the DNL algorithm. The distribution of the number of signal peaks with SNR setting equal to 2.0 for the spectra is shown in Figure 5. It can be seen that the distribution for the unidentified spectra was very well separated from that for the spectra with positive matches from the database search program. The majority of the unidentified spectra had fewer than 8 signal peaks, while most of the spectra with positive peptide matches had greater than 8 signal peaks. Figure 6 displays the histograms of the number of the spectra for these three categories, i.e. spectra with TPs, spectra with FPs and unidentified spectra, before and after DNL spectral screening. Overall 86.23% of the unidentified spectra and 27.33% of the spectra identified with FPs were removed by DNL spectral screening due to having fewer than 8 signal peaks. However, only 5.94% of the spectra with TPs were removed. In summary, the algorithm was able to screen out 86.23% of the unidentified spectra while keeping 94.06% of the spectra with TPs. After DNL spectral screening, the total percentage of the unidentified spectra in the data set was lowered from 80.87% to 41.06%, whereas that of the spectra with TPs in the data increased from 9.70% to 33.64%.

**Figure 7:**
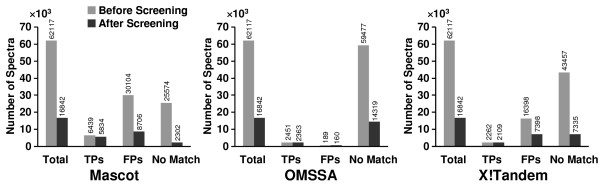
**Effect of DNL spectral screening on Mascot, OMSSA, and X!Tandem results**. Histograms showing the effect of DNL spectral screening on tandem mass spectra for the spectra with TPs, FPs and the unidentified spectra based on peptide identifications from Mascot, OMSSA, and X!Tandem.

The algorithm was also evaluated by the data based on the database search results from Mascot, OMSSA, and X!Tandem search engines with SNR and nmin settings equal to 2.0 and 8, respectively. Figure 7 shows the effects of DNL spectral screening on the Mascot, OMSSA, and X!Tandem search results. For Mascot, DNL spectral screening was able to filter 91.00% of the unidentified spectra and 71.08% of the spectra identified with FPs while keeping 90.60% of the spectra with TPs. For OMSSA, the algorithm filtered 75.93% unidentified spectra and 15.34% spectra identified with FPs and kept 96.41% spectra identified with TPs. For X!Tandem, the algorithm filtered 83.12% unidentified spectra and 54.88% spectra identified with FPs, and kept 93.24% spectra identified with TPs.

**Figure 8:**
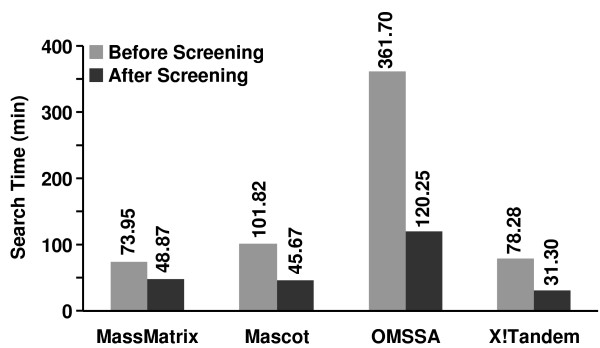
**Database search times of the data set before and after spectral screening**. Database search times of the data set against a protein database containing both the bovine histone database (117 proteins) and a decoy reversed human database (96,997 proteins) before and after spectral screening for MassMatrix, Mascot, OMSSA, X!Tandem. All searches were performed on a PC with Intel quad core CPU (2.4 GHz) and Linux operating system.

Due to the reduced number of spectra in the data set after DNL spectral, the database search times were also significantly reduced. As shown in Figure 8, the data search times for the merged data set for the four search engines were reduced by 33.92%-66.75%, or 25.08-241.45 min. Compared to the significantly reduced database search time, the spectral filtering time by use of DNL is trivial. It took the algorithm 0.78 min to filter the data set on a PC with Intel dual core CPU (2.4 GHz) and Windows XP operating system.

## Conclusion

A new dynamic noise level (DNL) algorithm has been developed to remove tandem mass spectra of poor quality. The algorithm was evaluated with a large data set that contained 62,117 spectra and was searched by MassMatrix against a database containing true protein sequences and a large decoy database. The algorithm determined the noise level dynamically and independently for each spectrum in tandem MS data. The distribution of noise in the spectra from the large test data set showed that the noise levels for tandem mass spectra varied significantly from one to another for ion trap mass spectrometry data. The algorithm assessed the quality of spectra based on the number of signal peaks and filtered those with less than 8 signal peaks. It was found that 89.0% of unidentified spectra in the MassMatrix database search program were successfully filtered while only 6.0% of spectra with true positive matches were removed upon DNL spectral screening. The algorithm was also found very effective at removal of unidentified spectra (75.93%-91.00%) in other database search programs including Mascot, OMSSA, and X!Tandem at a small loss (3.59%-9.40%) true positive matches.

## Availability and Requirements

Project name: Dynamic Noise Level Algorithm.

Project home page: http://www.massmatrix.net/.

Operating systems: Windows.

Programming language: ANSI C++.

Other requirements: None.

License: None.

Any restrictions to use by non-academics: None.

## Authors' contributions

HX designed the algorithm and drafted the manuscript. MAF was the principle investigator of the project and revised the manuscript critically. Both authors have read and approved the final manuscript.

## Supplementary Material

Additional file 1 **ROC curves of the DNL spectral screening algorithm.** ROC curves of the DNL spectral screening algorithm with the setting of SNR equal to 2 for the 23 individual tandem MS data sets. Click here for file
