# Supplementary material for: A Dynamic Noise Level Algorithm for Spectral Screening of Peptide MS/MS Spectra
Source: BMC Bioinformatics. 2010 Aug 23;11:436. doi: 10.1186/1471-2105-11-436 (PMC2939612; doi:10.1186/1471-2105-11-436)

Supplementary Figure 1. ROC curves of the DNL spectral screening algorithm with the setting of SNR equal to 2 for the 23 individual tandem MS data sets.

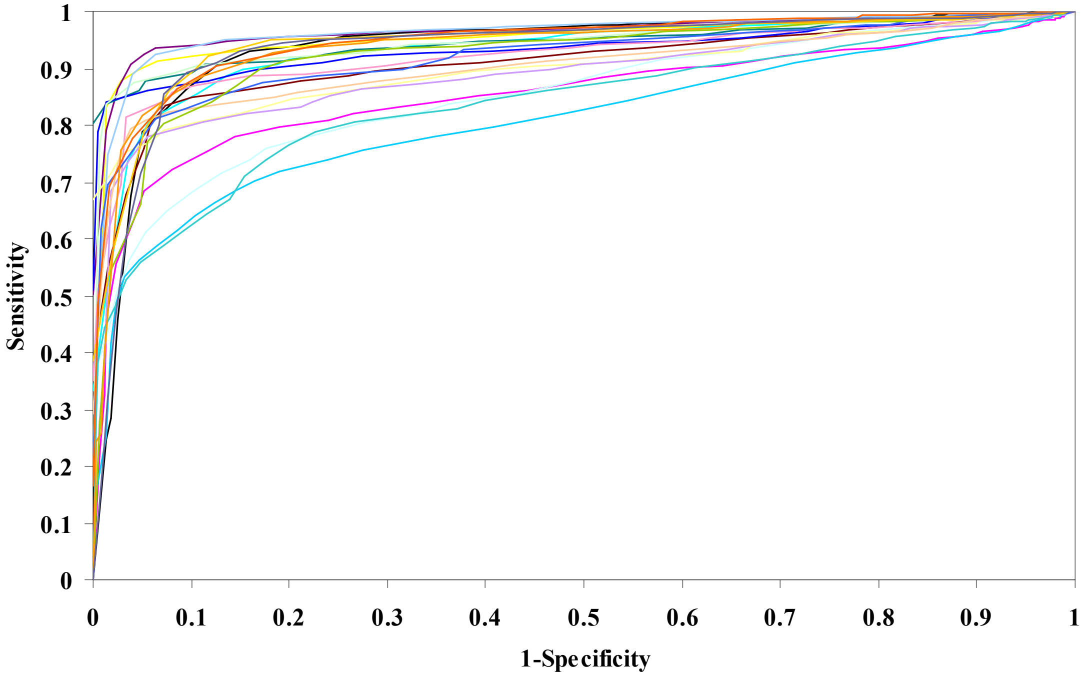

Supplement: Additional file 1 — ROC curves of the DNL spectral screening algorithm. ROC curves of the DNL spectral screening algorithm with the setting of SNR equal to 2 for the 23 individual tandem MS data sets. [file 1471-2105-11-436-S1.PDF]
